# Exploring correlation between preoperative gut microbiota and PONV using 16S absolute quantitative sequencing: a prospective observational study

**DOI:** 10.3389/fmed.2025.1563329

**Published:** 2025-05-26

**Authors:** Yijie Tang, Xiyuan Xie, Yu Guo, Yu Chen, Xinlei Huang, Dongsheng Dai, Xiaodan Wu

**Affiliations:** Department of Anesthesiology, Shengli Clinical Medical College of Fujian Medical University, Fujian Provincial Hospital, Fuzhou University Affiliated Provincial Hospital, Fuzhou, Fujian, China

**Keywords:** gut microbiota, 16S absolute quantitative sequencing, postoperative nausea and vomiting, *Bifidobacterium*, prevention

## Abstract

**Background:**

Postoperative nausea and vomiting (PONV) is a common complication following surgery. Despite various preventive measures, satisfactory outcomes have not been achieved. This study explores the potential of gut microbiota interactions with the host in understanding and preventing PONV, using 16S absolute quantitative sequencing technology to uncover new insights.

**Methods:**

Patients who experienced nausea and vomiting within 24 h after surgery were divided into a PONV group (*n* = 22) and a non-PONV group (*n* = 22). Microbial communities linked to PONV were assessed through bioinformatics analysis. Fecal samples from both groups were transplanted into rats, which were then anesthetized with isoflurane for 100 min. Pica behavior was monitored over the next 24 h to assess nausea and vomiting in the rats.

**Results:**

Significant differences in *α*- and *β*-diversity were observed between the PONV and non-PONV groups. Six key microorganisms were identified, with *Bifidobacterium*, *Bilophila*, and *Oscillibacter* showing a negative correlation with PONV severity. Receiver operating characteristic (ROC) analysis demonstrated that *Bifidobacterium* could reliably predict PONV. Rats receiving feces from the PONV group exhibited significantly higher kaolin consumption within 24 h post-anesthesia compared to those receiving feces from the non-PONV group.

**Conclusion:**

These results suggest a potential new mechanism for PONV involving gut microbiota, offering a theoretical basis for preoperative prediction of PONV based on gut microbial composition.

## 1 Introduction

Postoperative nausea and vomiting (PONV) is a common gastrointestinal issue that typically occurs within 24 h after surgery, affecting approximately 30% of general surgical patients and up to 80% in high-risk populations (1). Surveys have shown that vomiting and nausea are among the least tolerated postoperative reactions, with vomiting ranking first and nausea fourth in terms of patient discomfort (2). PONV negatively impacts patient comfort, recovery, and lengthens hospital stays (2). Despite various preventive measures, PONV continues to be inadequately controlled, highlighting the need for new approaches in its prevention.

The physiological mechanisms behind nausea and vomiting are complex, involving five main afferent pathways: (1) the central chemoreceptor trigger zone (CTZ) (3), (2) the vagus nerve in the gastrointestinal system (3), (3) neuronal pathways from the vestibular system (4), (4) the reflex afferent pathway of the cerebral cortex (5) and (5) the midbrain afferent pathway (6). Recent studies have proposed that the gut-brain axis may also play a role in PONV. One cohort study observed that patients undergoing esophagectomy and gastrectomy with vagotomy had a reduced risk of PONV, suggesting the vagus nerve-dependent gut-brain axis may contribute significantly to this condition (7). Additionally, research by Peng Cao and colleagues indicated that when the gastrointestinal tract is exposed to enterotoxins, enterochromaffin cells release serotonin, which signals the CTZ via the vagus nerve, triggering nausea and vomiting (8). However, the involvement of gut microbiota in PONV has yet to be explored.

Most microbiota analyses rely on relative quantitative sequencing, which only provides data on the relative abundance of microbial communities. This method can miss important information about the absolute number of microorganisms, potentially leading to misleading results (9, 10). Therefore, absolute quantitative sequencing was performed by adding a certain amount of artificial spiked-in reference standard sequences to the sample DNA as well as during amplicon library construction and when performing next-generation high-throughput sequencing. In contrast, absolute quantitative sequencing involves spiking the sample DNA with a reference standard, allowing for accurate calculation of microbial abundance based on standard curves and the absolute copy numbers of operational taxonomic units (OTUs)/amplicon sequence variants (ASVs) (11).

Various perioperative factors-such as emotions (12) (including anxiety and fear), sleep (13), antibiotics, stress (14), inhaled anesthetics (15) and opioids (16) can influence gut microbiota diversity. However, whether changes in microbiota composition contribute to PONV remains unclear. Therefore, this study aims to investigate the potential relationship between gut microbiota composition and PONV by assessing microbial diversity and abundance before surgery.

## 2 Materials and methods

### 2.1 Patients

This was a prospective, observational clinical study involving patients with thyroid cancer undergoing radical surgery admitted to the Fujian Provincial Hospital from June 2020 to February 2021. This study was approved by the Ethics Review Committee of Fujian Provincial Hospital (K2019-12-019, December 24, 2019) and written informed consent was obtained from all subjects participating in the trial. The trial was registered prior to patient enrollment at chictr.org.cn (ChiCTR2000029084, Principal investigator: Yi-Jie Tang and Xiao-dan Wu, Date of registration: January 13, 2021). Female patients aged 18–65 years, with American Society of Anesthesiologists (ASA) I-II status, not undergoing chemoradiotherapy, and scheduled for radical thyroid cancer surgery were included. Exclusion criteria were: (1) body mass index (BMI) > 28 kg/m^2^, (2) use of antibiotics or microecological regulators within 2 months prior to surgery, (3) absence of a preoperative fecal sample, (4) history of cognitive disorders, digestive diseases, allergies to anesthetics, or serious illnesses that may affect the intervention, outcomes, or ethical safety of the study (including heart failure, severe coronary artery disease and hypertension, chronic obstructive pulmonary disease, and hepatic or renal dysfunction), (5) vestibular labyrinthine dysfunction. All participants provided informed consent and agreed to take part in the study.

### 2.2 Fecal sample collection and clinical data

The study researcher, specifically trained for this purpose, guided patients in fecal sample collection and defecation 1 day before surgery. A trained clinical anesthesiologist was responsible for gathering data during the patients’ preoperative and postoperative visits, as well as conducting scale assessments.

#### 2.2.1 Sample collection

Patients were provided with disposable fecal collection boxes. Before defecation, they were asked to empty their bladder. A sterile spoon was used to collect the portion of feces that had not come into contact with the air or the collection box, and it was placed in a disposable sterile storage tube. After collection, the specimen tubes were immediately frozen in liquid nitrogen for 2 h, then transferred to a freezer for storage at −80°C.

#### 2.2.2 Assessment for nausea/vomiting and the basis for groupings

Nausea was defined as an unpleasant sensation accompanied by the awareness of the urge to vomit, while vomiting was described as the forceful expulsion of stomach contents through the mouth. The severity of nausea and vomiting was assessed using the World Health Organization (WHO) classification (17): 0, no nausea or vomiting; 1, nausea without vomiting; 2, vomiting 1–2 times per day; 3, vomiting 3–5 times per day, requiring pharmacological control; 4, vomiting more than six times per day, difficult to control with medications.

Patients were evaluated for the severity of nausea and vomiting 24 h after surgery. Those with a score ≥1 were placed in the PONV group, while the remaining patients were categorized into the non-PONV group. If the score was ≥3, patients received 5 mg of intravenous ropivacaine for antiemetic treatment.

#### 2.2.3 Demographic and clinical variables

The Self-Rating Anxiety Scale (SAS) was used to assess the preoperative anxiety level of patients (18). The Pittsburgh Sleep Quality Index (PSQI) measured baseline subjective sleep quality 24 h before surgery (19). Additionally, the St. Mary’s Hospital Sleep Questionnaire (SMHSQ) was employed to record subjective sleep quality during the same period (20). Rest pain intensity was evaluated 24 h post-surgery using the Visual Analog Scale (VAS) (21). Information such as age, BMI, Apfel score (22), and other relevant details were recorded during the patients’ preoperative visits.

### 2.3 General anesthesia

Upon entering the operating room, patients were routinely monitored for invasive arterial blood pressure, heart rate, oxygen saturation, and respiratory rate. The depth of anesthesia was monitored using a BIS EEG Vista anesthesia depth monitor. Anesthesia induction included 0.02 mg/kg midazolam, 0.5 μg/kg sufentanil, and 0.15 mg/kg cisatracurium, along with a target-controlled infusion of propofol, set to a plasma target concentration of 3.0 μg/mL. During anesthesia maintenance, the target concentration of propofol was maintained at 3–6 μg/mL, while the remifentanil effect chamber concentration was kept between 4 and 6 ng/mL, and BIS values were maintained between 40 and 60. If intraoperative blood pressure dropped by more than 20% from baseline or heart rate fell below 40 beats/min, ephedrine or atropine was administered to stabilize the patient’s blood pressure and heart rate. Postoperatively, 0.25% ropivacaine was used for local infiltration analgesia, and patients with VAS scores ≥4 were given 100 mg of flurbiprofen axetil for additional pain relief.

### 2.4 16S absolute quantitative sequencing

Genomic DNA was extracted and amplified from the fecal samples. Sample DNA (10 ng/μL) and spike-in internal reference DNA were used as templates for PCR amplification, both added to the same PCR system. An appropriate proportion of spike-in reference DNA was included in each sample for the target region, and a positive control was also prepared. DNA libraries were constructed, and sequencing was conducted using the Illumina NovaSeq system (Genesky Biotechnologies Inc., Shanghai, China).

### 2.5 Animals

Twelve male Sprague–Dawley rats, aged 6 weeks, were obtained from the Laboratory Animal Center of Fujian Medical University. They were housed in a clean laboratory at 24 ± 2°C with 50–70% humidity, simulating a standard 12-h light/dark cycle (ZT0-ZT12 light, ZT13-ZT24 dark), and had ad libitum access to food and water. The rats acclimated for 1 week prior to the experiment. All procedures were approved by the Experimental Animal Ethics Review and Use Committee of Fujian Medical University (IACUC FJMU 2022-0889) and followed the institution’s animal welfare regulations.

### 2.6 Fecal microbiota transplantation

Initially, antibiotics (ABX) were administered to both groups of fecal microbiota transplantation (FMT) recipient rats to create a pseudo germ-free model. The antibiotic cocktail, consisting of ampicillin sodium (1 mg/mL), neomycin sulfate (1 mg/mL), metronidazole (1 mg/mL), and vancomycin hydrochloride (0.5 mg/mL) (23), was given to the rats for 7 days after preparation. Fecal pellets were pooled, homogenized with 1 mL PBS and 1 mL skimmed milk per pellet (0.2 g) (24), and allowed to settle for 5 min. The supernatant (1 mL) was then administered to the rats via oral gavage daily for 14 days. Based on the source of the fecal solution, rats were randomly divided into two groups: ① FMT^non-PONV^ group, receiving feces from the non-PONV group; ② FMT^PONV^ group, receiving feces from the PONV group. The recipients were gavaged daily with the microbiota and were sacrificed after 2 weeks of FMT.

### 2.7 Anesthesia for animals

Rats were anesthetized in an induction chamber with 3% isoflurane. Once they lost the righting reflex, the isoflurane concentration was reduced to 1.5% for maintenance over 100 min. An isoflurane-specific calibrated vaporizer was used to administer 50% oxygen at a flow rate of 3 L/min (15). The loss and recovery of the righting reflex (LORR and RORR) were used as indicators of consciousness. LORR was defined as the inability of the rat to right itself within 30 s, while recovery was confirmed when the rat could turn onto all four limbs (25). After regaining the righting reflex, the rats were returned to their cages.

### 2.8 Rat pica model

Rats lack a central nervous system mechanism for controlling nausea and vomiting, which is primarily observed through pica behavior (26, 27). Kaolin was placed in a separate compartment of the food hopper 3 days before anesthesia to allow the rats to acclimate to its presence. Kaolin and food intake were measured 24 h after anesthesia. Following the experiment, the rats were housed in a clean laboratory at the Laboratory Animal Center of Fujian Medical University.

### 2.9 Statistical analysis

Statistical analyses of clinical data were conducted using SPSS 26.0 software (IBM Corp., Armonk, New York, United States). Descriptive statistics are presented as means ± standard deviations or percentages. Normality was assessed with the Shapiro–Wilk test, and normally distributed data were analyzed using the independent-samples t test. Non-normally distributed data are presented as the median [IQR] and analyzed using the Mann–Whitney U test. Correlation analysis was performed using Pearson’s correlation. Strain composition, *α*-diversity, *β*-diversity, and functional analyses were conducted using QIIME (V1.9.1) and R packages (ggplot2 [3.3.6], stats [4.2.1], car [3.1–0], vegan [2.6.4], and ape [5.6.2]). *p*-value < 0.05 was considered statistically significant.

## 3 Results

### 3.1 Demographic data

**Figure 1:**
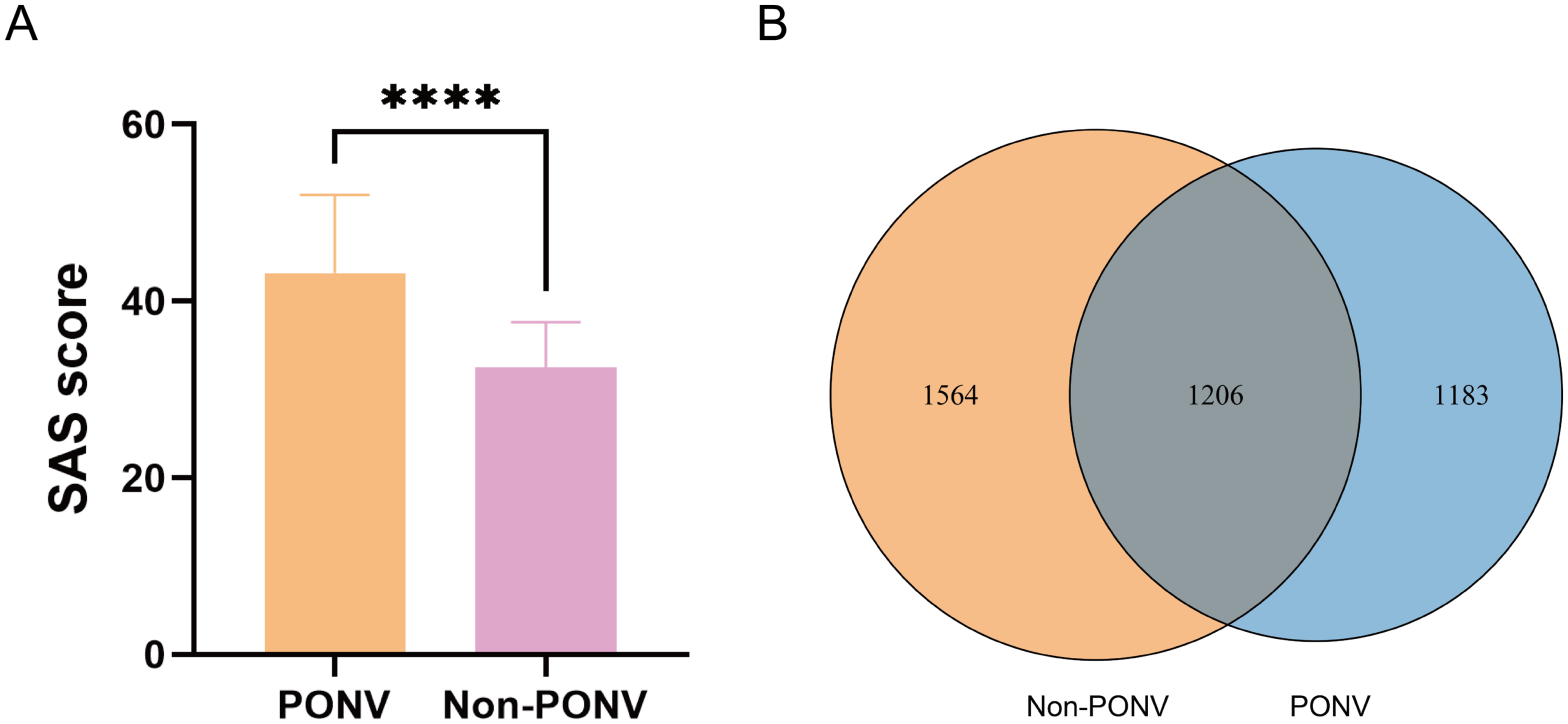
**(A)** Compared with the non-PONV group, the mean SAS score was significantly higher in the PONV group (non-PONV group vs. PONV group: 32.55 ± 5.09 vs. 43.18 ± 8.86, respectively, *p* < 0.0001). **(B)** As shown in the Venn diagram, a relatively low number of shared OTUs between the two groups, with 1,206 OTUs. The PONV group had a significantly lower gut microbiome abundance compared to the non-PONV group, with 2,389 OTUs.

The study adhered to Strengthening the Reporting of Observational Studies in Epidemiology (STROBE) guidelines. As shown in Supplementary Figure 1, 82 patients were assessed for eligibility, and 72 were included in the study. Ten patients were excluded due to the lack of fecal samples, four had received antibiotics, six had gastrointestinal diseases, and eight refused to participate. Ultimately, 44 patients were included, with 22 assigned to the PONV group (experiencing nausea and vomiting) and the remaining patients assigned to the non-PONV group. The PONV group had a significantly higher mean SAS score compared to the non-PONV group (non-PONV: 32.55 ± 5.09, PONV: 43.18 ± 8.86, *p* < 0.001) (Figure 1A). There were no significant differences in other demographic factors, including age, BMI, Apfel score, PSQI score, St. Mary’s sleep questionnaire, VAS scores, and the duration of the operation, anesthesia, recovery, or the administration of sufentanil and propofol (Table 1).

**Table 1:** The demographic data of the patients in this study.

Variables	PONV (*n* = 22)	Non-PONV (*n* = 22)	*p*-value
Age (years), mean ± SD	46.23 ± 12.57	44.09 ± 8.22	0.509
BMI, mean ± SD	22.13 ± 2.43	23.14 ± 2.16	0.154
Apfel score, *n* (%)			1.000
2	12 (27.30%)	12 (27.30%)	
3	10 (22.70%)	10 (22.70%)	
SAS score, mean ± SD	43.18 ± 8.86	32.55 ± 5.09	<0.001^***^
PSQI score, mean ± SD	7.18 ± 4.14	5.68 ± 3.34	0.239
St. Mary’s sleep questionnaire, mean ± SD	23.72 ± 6.10	25.86 ± 6.74	0.277
Operation duration (min), mean ± SD	80.05 ± 23.65	86.27 ± 34.95	0.493
Anesthesia duration (min), mean ± SD	100.09 ± 22.19	107.59 ± 34.36	0.395
Recovery duration (min), mean ± SD	7.77 ± 4.75	7.68 ± 5.52	0.954
Sufentanil (ug), mean ± SD	30.00 ± 4.88	33.18 ± 6.46	0.073
Propofol (mg), mean ± SD	401.32 ± 95.51	426.09 ± 164.18	0.545
VAS score, mean ± SD	2.82 ± 1.14	2.55 ± 0.86	0.375

PONV, postoperative nausea and vomiting; *n*, sample size; SD, standard deviation; BMI, body mass index; SAS, Self-Rating Anxiety Scale; PSQI, Pittsburgh Sleep Quality Index; VAS, visual analog scale; ^***^p < 0.01.

### 3.2 Diversity analysis of the gut microbiota between the groups

**Figure 2:**
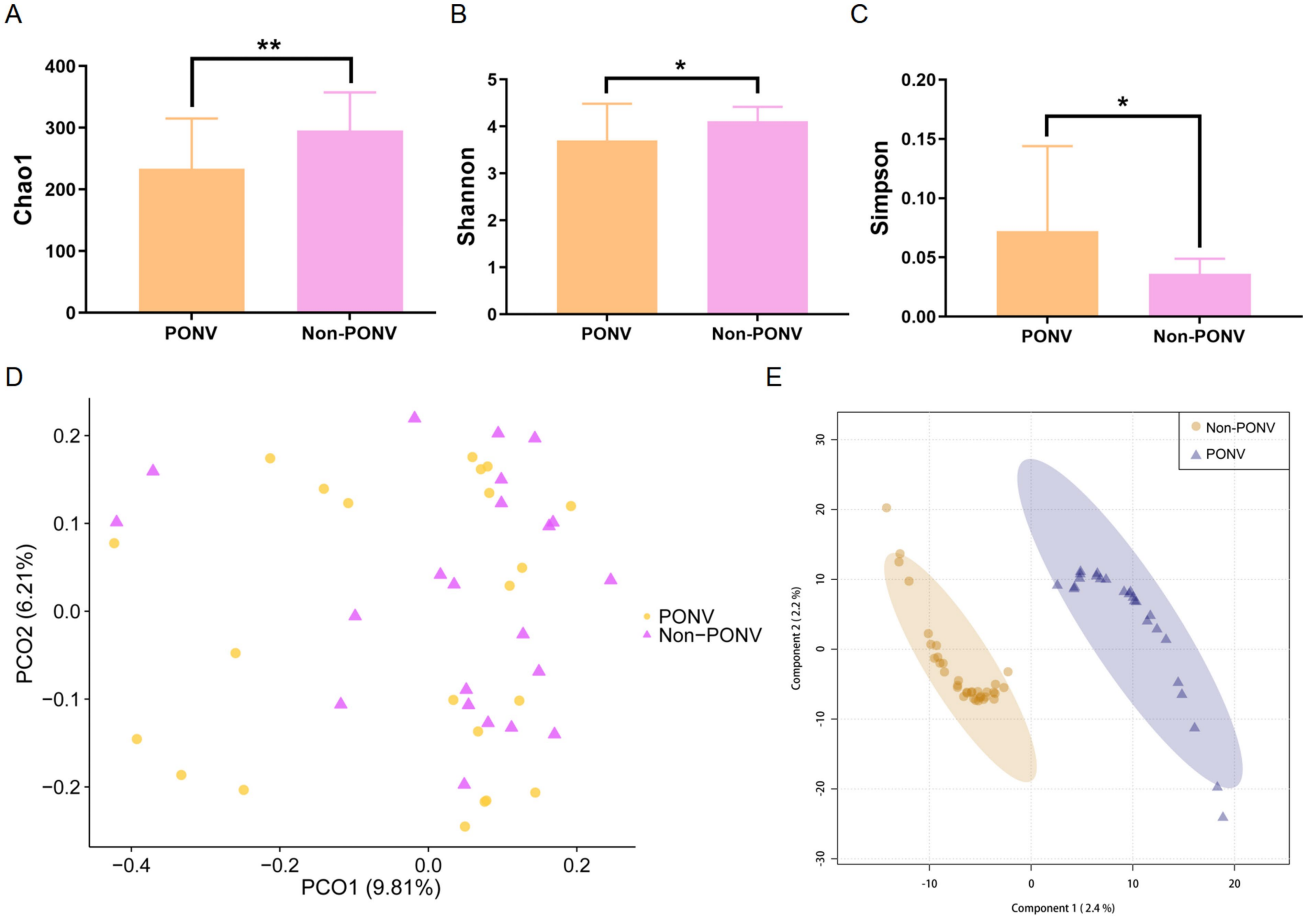
**(A–C)** *α*-diversity between the two groups as estimated by the Chao1, Shannon and Simpson indexes (*p* = 0.0071, *p* = 0.0283, *p* = 0.0254, respectively). **(D,E)** *β*-diversity reflecting the significant differences in community composition between the two groups (*p* = 0.0495).

The OTU Venn diagram revealed a relatively low number of shared OTUs between the two groups, with 1,206 OTUs. The PONV group had a significantly lower gut microbiome abundance compared to the non-PONV group, with 2,389 OTUs (Figure 1B). The *α*-diversity index, which measures species abundance and diversity, showed that the PONV group had significantly lower Chao1 and Shannon indexes, and a significantly higher Simpson index, compared to the non-PONV group (Figures 2A–C). These results indicate significant differences in α-diversity between the two groups, with lower microbiome abundance and diversity in the PONV group. To analyze *β*-diversity, principal coordinate analysis (PCoA) and partial least squares-discriminant analysis (PLS-DA) were performed, and PERMANOVA was applied. The results revealed significant differences in microbial community composition between the groups, with a dispersed distribution (*p* = 0.0495; Figures 2D,E).

### 3.3 Composition and absolute abundance of gut microbiota between the groups

**Figure 3:**
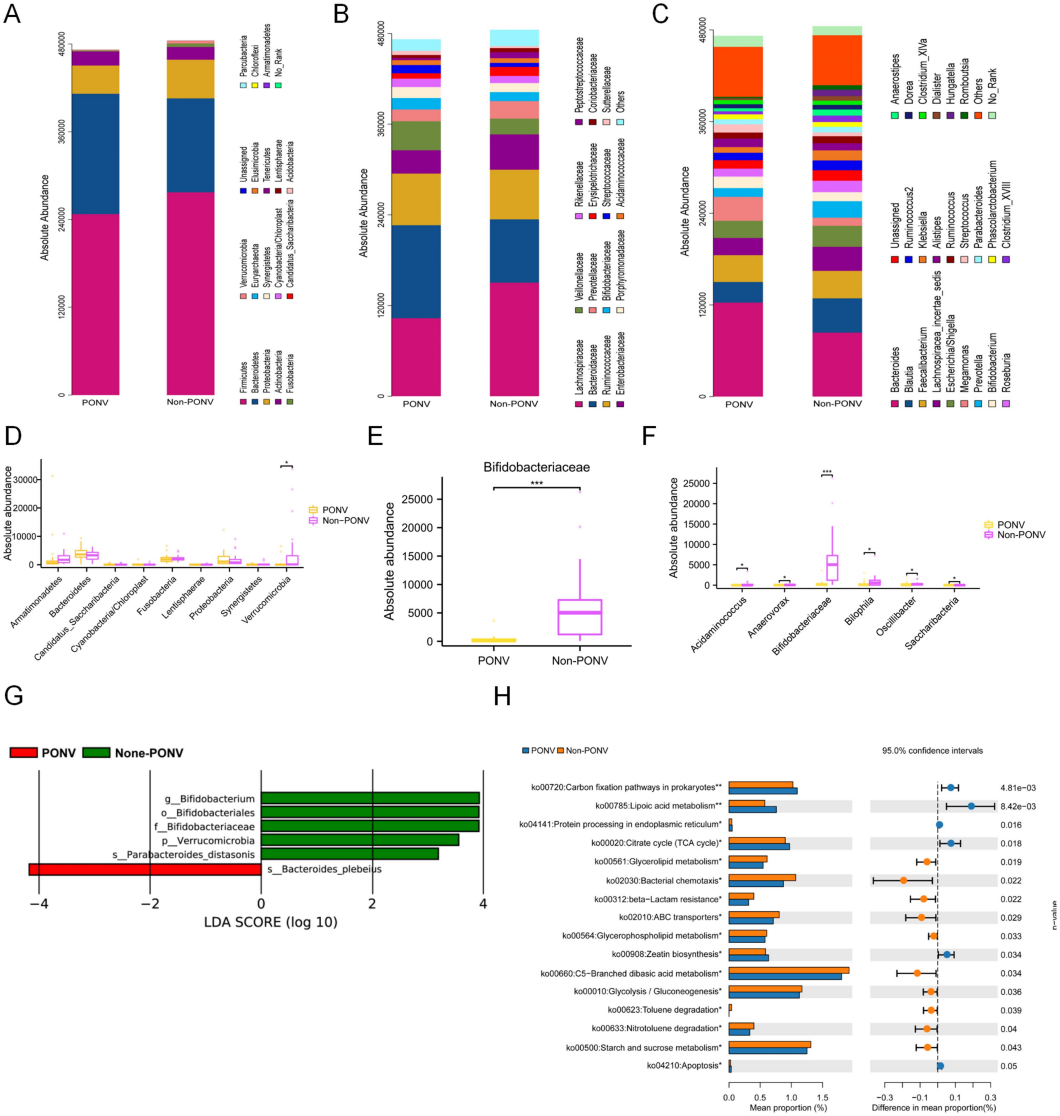
**(A–F)** The significant differences in gut microbiota between the two groups. At the phylum level, the PONV group had a lower absolute abundance of Verrucomicrobia than the non-PONV group. At the family level, the absolute abundance of Bifidobacteriaceae in the PONV group was lower than that in the non-PONV group. At the genus level, the non-PONV group had a higher absolute abundance of *Acidaminococcus*, *Anaerovorax*, *Bifidobacterium*, *Bilophila*, *Oscillibacter* and *Saccharibacteria.* **(G)** According to the LDA score size, the phylum Verrucomicrobia, the order Bifidobacteriales, the family Bifidobacteriaceae and the genus *Bifidobacterium* as well as the species *Bacteroides plebeius* and *Parabacteroides distasonis* may be the main biomarkers that lead to the difference between the PONV and non-PONV groups. **(H)** Enrichment of the different functions between the two groups. ^*^p < 0.05, ^**^p < 0.01, ^****^p < 0.0001.

At the phylum level, the PONV group exhibited a lower absolute abundance of *Verrucomicrobia* (*p* = 0.036) compared to the non-PONV group (Figures 3A,D). At the family level, *Bifidobacteriaceae* abundance was also lower in the PONV group than in the non-PONV group (*p* < 0.001) (Figures 3B,E). At the genus level, the non-PONV group showed higher levels of *Acidaminococcus*, *Anaerovorax*, *Bifidobacterium*, *Bilophila*, *Oscillibacter*, and *Saccharibacteria* (Figures 3C,F). These differences were further validated by LEfSe analysis using linear discriminant analysis (LDA), which suggested that *Verrucomicrobia* (phylum), *Bifidobacteriales* (order), *Bifidobacteriaceae* (family), *Bifidobacterium* (genus), and *Bacteroides plebeius* and *Prabacteroides* distasonis (species) may serve as key biomarkers differentiating the PONV and non-PONV groups (Figure 3G). Functional enrichment analysis revealed that the microbiota in the PONV group were significantly associated with pathways like lipoic acid metabolism, nitrotoluene degradation, and apoptosis (Figure 3H).

### 3.4 Correlation analysis of gut microbiota and clinical data

**Figure 4:**
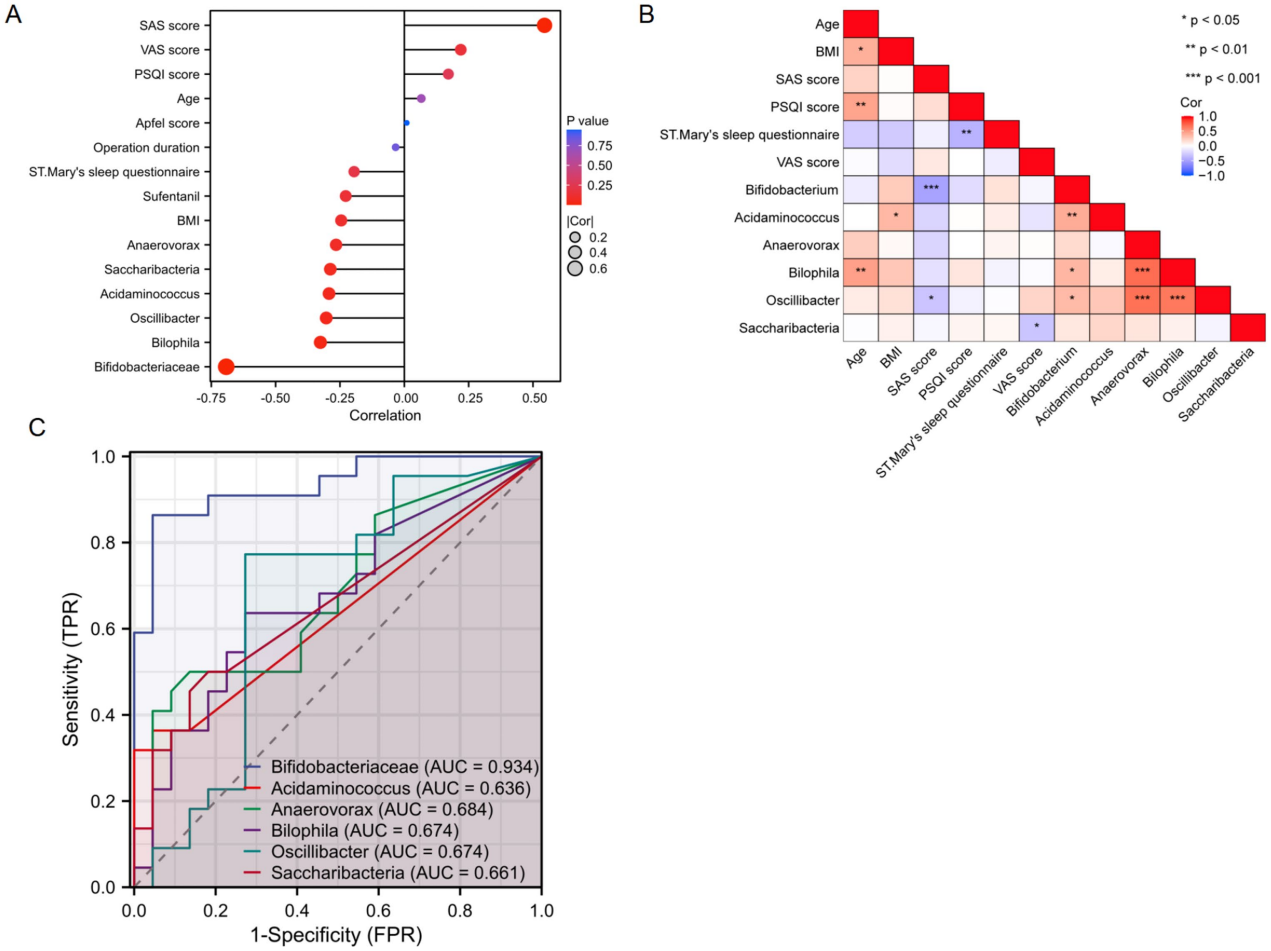
**(A,B)** Correlation analysis between the gut microbiota and clinical indicators. **(C)** ROC analysis of representative differential gut microorganisms for predicting PONV.

The SAS score showed a positive correlation with the severity of PONV (r = 0.5448, *p* < 0.001). In contrast, the genera *Bifidobacterium*, *Bilophila*, and *Oscillibacter* were negatively correlated with PONV severity (*r* = −0.6919, *p* < 0.001; *r* = −0.3259, *p* = 0.030; *r* = −0.3034, *p* = 0.045, respectively; Figure 4A). *Bilophila* was positively correlated with age (*r* = 0.4594, *p* = 0.002), and *Acidaminococcus* was positively correlated with BMI (*r* = 0.3471, *p* = 0.021). *Bifidobacterium* and *Oscillibacter* had negative associations with the SAS scores (r = −0.4943, *p* < 0.001; r = −0.3099, *p* = 0.041, respectively), while *Saccharibacteria* was negatively associated with the VAS score (r = −0.3074, *p* = 0.042; Figure 4B). No correlations were found between age, BMI, Apfel score, PSQI score, St. Mary’s sleep questionnaire score, operation duration, sufentanil, VAS score, and the abundances of *Acidaminococcus*, *Anaerovorax*, and *Saccharibacteria* with PONV severity.

### 3.5 Potential predictive functions of gut microbiota

The predictive ability of representative gut microbiota for PONV was assessed using the receiver operating characteristic (ROC) curve, a common analytical method for evaluating model performance. The results indicated that *Bifidobacterium*, a key differential microbiota, could effectively predict PONV (AUC = 0.934, *p* < 0.01; Figure 4C).

### 3.6 PONV is mediated by gut microbiota in rats

**Figure 5:**
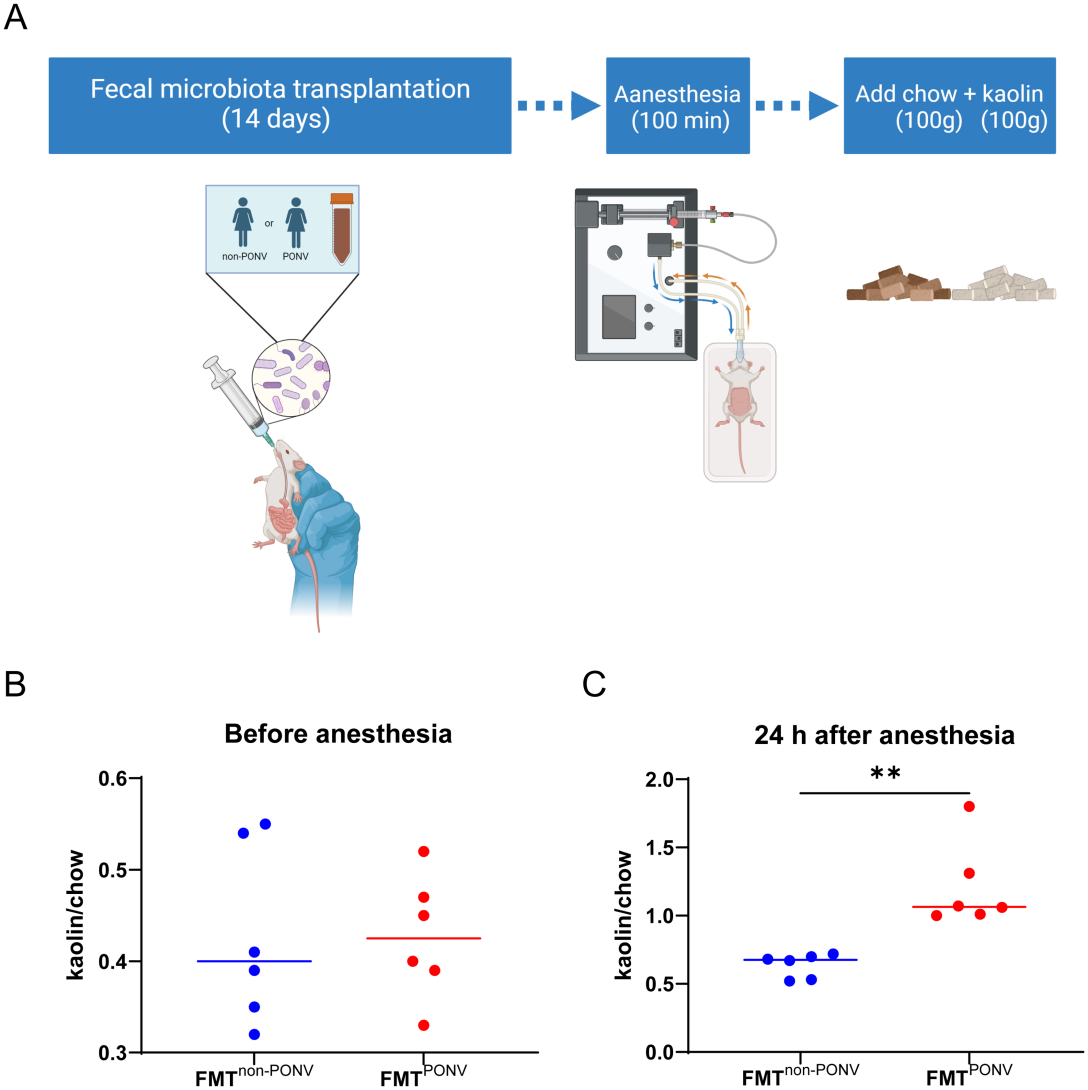
**(A)** Procedures for animal experiments; **(B)** The level of kaolin intake was similar between the two groups before anesthesia. **(C)** Rats that received feces from patients in the PONV group consumed significantly greater amounts of kaolin within 24 h after anesthesia compared to those received feces from patients in the non-PONV group.

Animal experiments were needed to verify that gut microbiota is one of the potential mechanisms of PONV. As shown in Figure 5A, fecal samples from PONV and non-PONV patients were transplanted into the gut tract of the rats. After 14 days of colonization, the rats were anesthetized with isoflurane for 100 min. Pica behavior was observed within 24 h after anesthesia to reflect nausea and vomiting of rats. The level of kaolin intake was similar between the two groups before anesthesia (Figure 5B). Rats that received feces from patients in the PONV group consumed significantly greater amounts of kaolin within 24 h after anesthesia compared to those received feces from patients in the non-PONV group (Figure 5C).

## 4 Discussion

This study explored the connection between gut microbiota and PONV, revealing significant differences between female patients with and without PONV. We found a notable reduction in microbiota richness in PONV patients, along with distinct differences in community composition compared to non-PONV patients. Currently, no similar studies have been reported. Through fecal microbiota transplantation experiments, it was confirmed that the gut microbiota is a potential mechanism for the occurrence of PONV.

This study found that patients with PONV had higher anxiety scores compared to those without PONV. Several prospective studies have shown a link between preoperative anxiety and PONV, with heightened anxiety potentially serving as a predictor for PONV risk (28). Reducing anxiety and improving sleep quality could also help lower PONV incidence (29). Roh et al. (30) similarly found that patients who experienced PONV had higher preoperative anxiety scores. These findings align with our results. Previous research also indicated that PONV patients had higher Apfel scores (22), longer surgery durations (31) and greater use of sufentanil (32) than those without PONV. The discrepancies in our study may be attributed to the following factors: (1) The Apfel score was based on four independent risk factors: female sex, history of PONV and/or motion sickness, nonsmoking status, and postoperative opioid use. Due to our study’s inclusion criteria, all participants were nonsmoking females who did not use opioids post-surgery, and (2) all patients in our study underwent radical, straightforward thyroid cancer surgeries, with no significant difference in operation time between the two groups.

*Bifidobacterium*, a genus that mainly produces SCFAs such as acetic acid and lactic acid, which can improve gastrointestinal function, was negatively associated with the severity of PONV and the state of anxiety in our study. 5-HT enhances anxiety by acting on forebrain structures (33). Supplementation with Bifidobacterium reduced plasma and central 5-HT levels in individuals (34). The abundance of Bifidobacterium in people (35) or mice (36) with anxiety and depression is significantly reduced, which is consistent with findings from this study. Therefore, we consider that patients with a reduced abundance of Bifidobacterium are more likely to develop PONV, which may be related to the improvement of anxiety state by 5-HT reduction. *Oscillospira* is a gram-positive bacterium. From the metagenomic and metabolic profiles, the finding that *Oscillospira* possesses a butyrate kinase-mediated pathway can produce butyric acid (37). A variety of exogenous factors affect the abundance of *Oscillospira,* such as probiotics, heavy metals, naturally active products, pharmacological interventions, exercise and diet and diseases (38). The genus was found to be negatively associated with preoperative symptoms of anxiety in the present study, which is consistent with the results of many studies. *Oscillibacter* was also found to be negatively associated with the severity of PONV, and this may be related to the reuptake of 5-HT caused by these organisms (39). We performed functional prediction analysis for different species and found that the involvement of the lipoic acid metabolic pathway in PONV may be related to 5-HT. This further supported our previous conjecture. Freitas et al. (40) found that lipoic (also known as thioctic) acid, which inhibited the hyperactivity of the 5-HTergic neuronal system during pilocarpine-induced seizures, could normalize the levels of dopamine (DA) and 5-HT (39). Another study found that coadministration of lipoic acid and a 5-HT reuptake inhibitor improved anxiolytic and antidepressant responses (41). However, further studies are needed to clarify the role of the lipoic acid pathway in PONV.

Our study found that the preoperative anxiety state was positively correlated with the severity of PONV. Previous studies found a correlation between preoperative anxiety and PONV, but the correlation was weak (42), which was consistent with our results. However, another study found no correlation between anxiety and PONV (43). The differences in the above results may be due to the differences in anxiety assessment and anesthesia levels as well as medication and many other factors. Many studies suggest that the susceptibility to motion sickness decreases with age, so patients younger than 50 years old were considered to be more likely to develop PONV (44). Our study, however, found no correlation between age and the occurrence of PONV. This discrepancy may be due to the fact that the patients in our study were primarily from medium- and high-risk populations, where age might not have a significant impact on the development of PONV.

FMT is an extremely important and highly effective experimental approach for investigating host–microbe interactions. The humanized mouse model, which is established by transplanting single bacteria, multiple bacteria or flora into germ-free (GF) mice, or by transplanting human feces into GF mice after treatment, can achieve the transfer and translocation of intestinal flora between different animals. Subsequently, it can be used to explore the causal relationship between intestinal flora and disease phenotype, thus serving as an important method for studying the relationship between the human microbiome and disease (45, 46). In animal experiments, GF mice are ideal recipients for microbiota transplantation. By introducing specific gut microbiota at different developmental stages of GF mice, the underlying mechanisms and their effects on health and disease can be explored. However, the high price of sterile animals, strict feeding environment and technical requirements make it difficult to carry out experimental operation in ordinary laboratories. Therefore, most researchers tend to choose ABX, which has the effect of eliminating gut microbes and is cheap, to construct pseudo-germ-free mice to study the role of gut microbiota (47). As a result, many researchers use antibiotics (ABX) to create pseudo-germ-free mice, as they are more affordable and effective in eliminating gut microbes. Studies have shown that broad-spectrum ABX “cocktails” can eliminate up to 90% of intestinal microorganisms in mice (47, 48). In this study, pseudo-sterile rats were used as recipients for fecal transplants from patients in both the non-PONV and PONV groups. The results showed that pica occurred within 24 h after anesthesia in the FMT^PONV^ group, effectively replicating the disease phenotype of postoperative nausea and vomiting.

TDespite the promising findings, our study had some limitations. Clinical trials to validate the role of *Bifidobacterium* in predicting and managing PONV were not conducted, and we plan to address this in future research. This study is based on a small sample size, which may not fully capture inter-individual variability and lacks population representativeness. Further validation with larger cohorts is needed to explore the underlying mechanisms and identify potential intervention targets in greater depth. Additionally, the study controlled for patient sex and surgical type, meaning the results are specific to nonsmoking female patients undergoing radical thyroid cancer surgery. Future studies should explore these mechanisms in more depth. Nonetheless, this preliminary study provides initial evidence of a link between gut microbiota and PONV.

## 5 Conclusion

The gut microbiota of patients in the non-PONV and PONV groups was analyzed using 16S absolute quantitative sequencing. The findings revealed significant differences in gut microbiota between the two groups, with six key microbial species showing distinct correlations with PONV occurrence. ROC curve analysis further demonstrated that *Bifidobacterium* could potentially predict PONV versus non-PONV status. The FMT experiment suggested a novel mechanism for PONV from the perspective of gut microbiota, providing a theoretical basis for predicting PONV based on preoperative gut microbiota. This aligns with the medical concept of enhanced recovery after surgery.

## Data Availability

The datasets presented in this study can be found in online repositories. The names of the repository/repositories and accession number(s) can be found in the article/Supplementary material.
