# Supplementary material for: Exploring correlation between preoperative gut microbiota and PONV using 16S absolute quantitative sequencing: a prospective observational study
Source: Front Med (Lausanne). 2025 May 26;12:1563329. doi: 10.3389/fmed.2025.1563329 (PMC12146284; doi:10.3389/fmed.2025.1563329)
Supplement: Supplementary file 1 [file Data_Sheet_1.pdf]

Self-Rating Anxiety Scale (SAS)

Below are 20 statements. Please read each one carefully, understand their meanings, and then choose the " " that best matches your actual feelings on scales 1 to 4. Do not spend too much time thinking about each question; answer based on your first impression. For self-assessment of current primary emotional and physical symptoms, select according to the severity of your symptoms.

(Evaluation time is within the past week or now)

Evaluatio n project	None or Very few	Sometimes there is	Most Time is on our side (Frequently)	Mostly so There are a few minutes
1、 I feel more neurotic and anxious than usual	1	2	3	4
2、 I was worried for no reason	1	2	3	4
3、 I tend to get upset or feel panicked	1	2	3	4
4、 I felt as if my body was being split into pieces, sh-attered	1	2	3	4
*5. I feel that everything is going well and nothing bad will happen	4	3	2	1
6 、 My limbs tremble and quiver	1	2	3	4
7、 I am troubled by heada-ches, neck pain, back pain	1	2	3	4
8、 I feel weak and tired	1	2	3	4
I feel very calm and can sit quietly	4	3	2	1
10、 I felt my heart beat-ing faster	1	2	3	4
11 、 I was uncomfortable with the dizziness	1	2	3	4
12 、 I had a feeling of fainting	1	2	3	4
:: 13. I breathe without any effort to take in or expel air	4	3	2	1
14 、 My fingers and toes feel numb and tingling	1	2	3	4
15 、 I was suffering from stomach pains and indigestion	1	2	3	4
16 、 I have to go to the bathroom frequently	1	2	3	4
* My hands are always warm and dry	4	3	2	1

18、 I think my face is hot and red	1	2	3	4
I fall asleep easily and rest well at night	4	3	2	1
20、 I have nightmares	1	2	3	4

Scoring and interpretation:

1、 The evaluation adopts 1~4, and the score is calculated.

2、 Add up the scores of the 20 questions to get the total score, multiply the total score by 1.25, round it off to an integer, and you have the standard score.

3、 The cut-off value of anxiety rating was 50 points, mild anxiety was 50-59 points, moderate anxiety was 60-69 points, and severe anxiety was above 70 points. The higher the score, the more obvious the anxiety tendency.

I. Applicable objects

The SAS is suitable for adults with anxiety symptoms and, like the SDS, has a broader applicability.

Evaluation methods and matters needing attention

Before self-assessment, he should be made to understand the method of filling out the whole scale and the meaning of each question, and then make an independent self-assessment without being influenced by anyone.

Before starting the evaluation, the staff member will point to the SAS scale and tell him: There are twenty statements below. Please read each one carefully, understand its meaning, and then mark an appropriate checkmark (√) in the corresponding box based on your actual situation over the past week. Each statement is followed by four boxes, representing no or rarely (occurred), some of the time, quite a bit of the time, most of the time, or all of the time.

If the evaluator is too illiterate to understand or read the SAS questions, the staff can read them to him one by one and let the evaluator make his own evaluation independently. A single evaluation can generally be completed within ten minutes.

Note:

1.The time frame for evaluation should emphasize "now or the past week."

2 At the end of the evaluation, the staff should carefully check the self-evaluation results and remind the self-evaluator not to miss a project or to tick two ticks in the same project (i.e., do not repeat the evaluation).

3 The SAS should be rated once by the self-assessor before starting treatment, and then at least once after treatment (or at the end of the study) to analyze changes in symptoms through changes in the total SAS score. If ratings are made during treatment or the study, the intervals can be arranged by the investigator.

Results analysis

The primary metric for SAS is the total score. After self-assessment, the scores of all 20 items are added together to obtain the raw score (raw score). This raw score is then converted using the formula y = int(1.25x); that is, the raw score is multiplied by 1.25 and the integer part is taken, resulting in the standard score (index score, Y), or it can be converted through a table lookup.

It must be emphasized that in SAS, the scores of items 5, 9, 13,17 and 19 out of 20 items must be calculated in reverse.

Indications: (1) Repeated visits to relevant departments of general hospitals due to headaches, neck pain, back pain, lower back pain, and limb pain, with no organic lesions indicated by clinical examination and laboratory tests. (2) Chronic pain caused by mental factors such as anxiety, fear, hypochondria, and depression. (3) Chronic generalized pain of various causes. (4) Tension-type headache. (5) Migraine.

Contraindications: (1) patients with myocardial infarction or severe arrhythmia or heart failure during or after the onset of myocardial infarction.(2) Severe diseases of major organs, such as patients with liver and kidney dysfunction, respiratory failure, cerebral hemorrhage, cerebral infarction, and patients with unstable diabetes. (3) Schizophrenia in an active phase. (4) Severe intellectual disability, uncooperative with examinations.
