# Supplementary material for: Exploring correlation between preoperative gut microbiota and PONV using 16S absolute quantitative sequencing: a prospective observational study
Source: Front Med (Lausanne). 2025 May 26;12:1563329. doi: 10.3389/fmed.2025.1563329 (PMC12146284; doi:10.3389/fmed.2025.1563329)
Supplement: Supplementary file 3 [file Table_2.DOC]

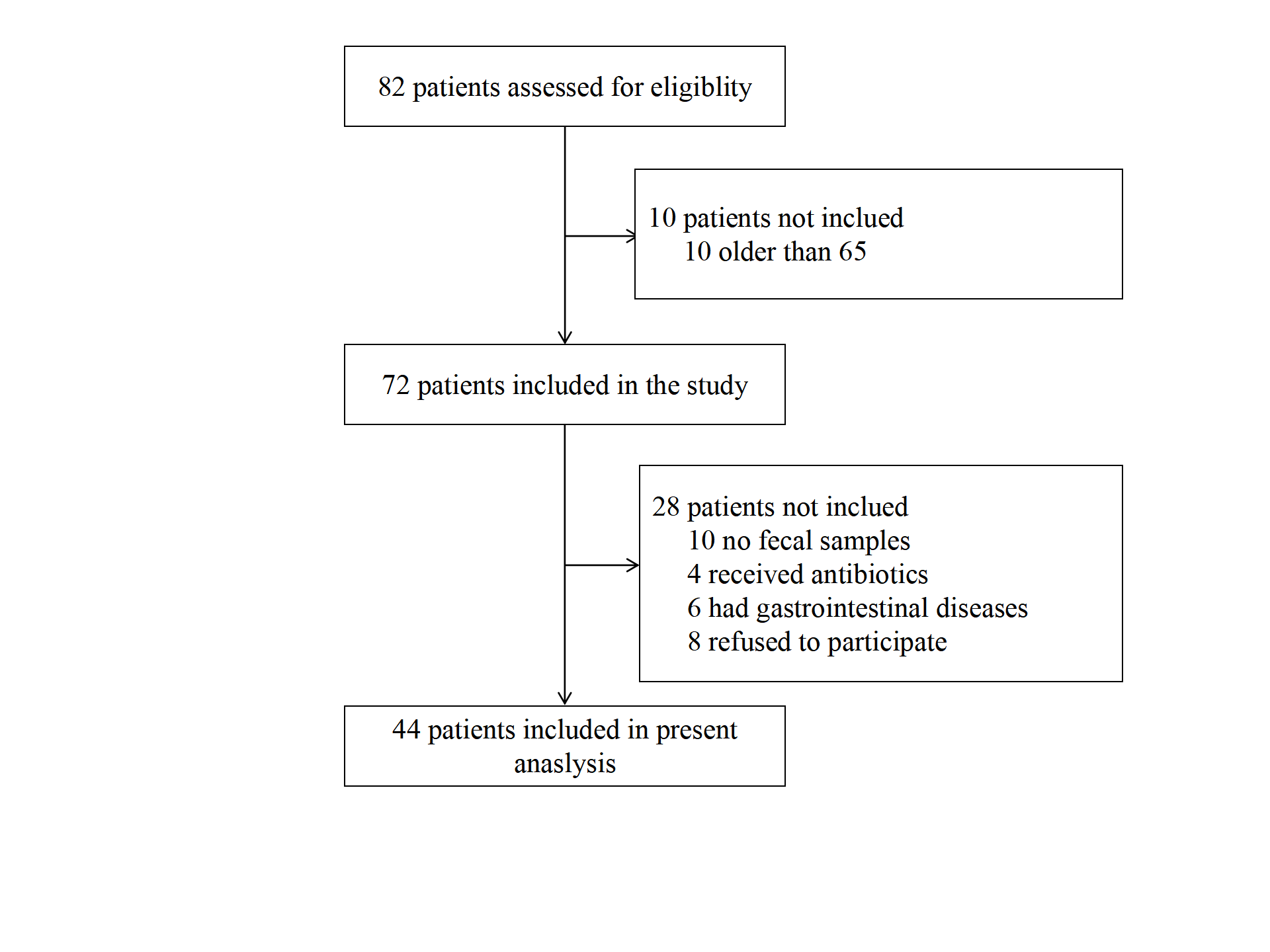


Supplemental Fig. 1 STROBE flow diagram of patient screening, inclusion, and analysis. The study was a prospective, observational study.
